# Long‐term seed bank persistence in a stochastic desert environment

**DOI:** 10.1002/ece3.9924

**Published:** 2023-03-21

**Authors:** Tara de Queiroz, Susan E. Meyer

**Affiliations:** ^1^ Shrub Sciences Laboratory USDA Forest Service Rocky Mountain Research Station Cedar City Utah USA

**Keywords:** *Arctomecon californica*, climate change, cue‐nonresponsive dormancy, germination, Las Vegas bearpoppy, Mojave Desert, rare plant

## Abstract

Seed banks, the collection of viable seeds in the soil, are particularly important determinants of population survival in highly variable environments. Predictions of increased stochasticity in the amount and timing of precipitation in desert environments raise the question of how seed banks of desert species will respond to climate change, and ultimately, whether these species will persist. Here, we present data from our long‐term studies of germination requirements and seed bank dynamics in a rare desert gypsophile perennial, *Arctomecon californica* (Las Vegas bearpoppy). *Arctomecon californica* is a relatively short‐lived plant that recruits from seed in sequences of unusually favorable years. We used germination experiments, an in situ seed bank study, and a 15‐year field seed retrieval study to examine factors affecting seed bank persistence. In the germination study, a majority of seeds remained dormant, despite a wide variety of treatments, suggesting that a large proportion of the seed dispersed each year has cue‐nonresponsive dormancy. Our in situ seed bank study showed that seed density varied widely between sites, among transects, and among samples within a transect. The patchiness of seeds in the soil highlights the importance of protecting large areas where *A*. *californica* populations are known to have existed in the past. The seed retrieval study provided strong evidence that this species has a long‐lived seed bank in which only a small fraction of seeds (roughly 5%) become nondormant each year, allowing seed banks of this species to last up to 20 years without a seed production event. Whether this impressive life‐history strategy can maintain the species in the face of climate change depends on the future frequency of the well‐timed precipitation that allows for the establishment of new cohorts of adult plants.

## Graphical abstract

*Arctomecon californica* is a short‐lived gypsophile perennial that is found in a stochastic desert environment. We demonstrated through field and laboratory studies that it can survive extended periods of failed recruitment through the maintenance of a persistent seed bank that can last up to 20 years. The seeds have cue‐nonresponsive dormancy that buffers the seed bank against rapid depletion so that populations that have not had actively growing plants for as long as two decades may still have the capacity to reestablish from the seed bank.

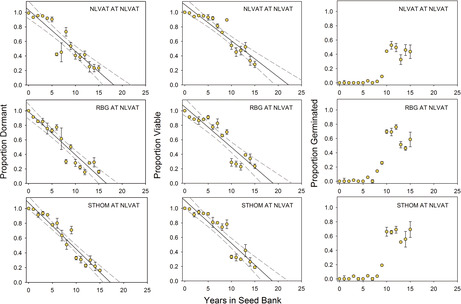

## 1 INTRODUCTION

In populations of annual plants, all aboveground individuals are in the same age class. For perennials, individuals can establish in different years, creating multiple age classes. However, both annual and perennial species can have another pool of individuals that are crucial to population persistence: the collection of dormant seeds that carry over across years to form the persistent seed bank (Fenner & Thompson, 2005).

For annual plants, a seed bank can ensure population persistence in years when recruitment fails (Venable, 2007). For perennials, a seed bank can allow populations to persist across a series of unfavorable years when aboveground plants suffer mortality and are not replaced by recruitment (Van Buren et al., 2021). Seed banks are particularly important in highly variable environments, where the occurrence of favorable conditions is unpredictable (Cohen, 1966). They provide a classic example of the evolution of adaptive bet‐hedging (Gremer & Venable, 2014) that is particularly effective when seeds have high survival (Cuello et al., 2019).

**FIGURE 1:**
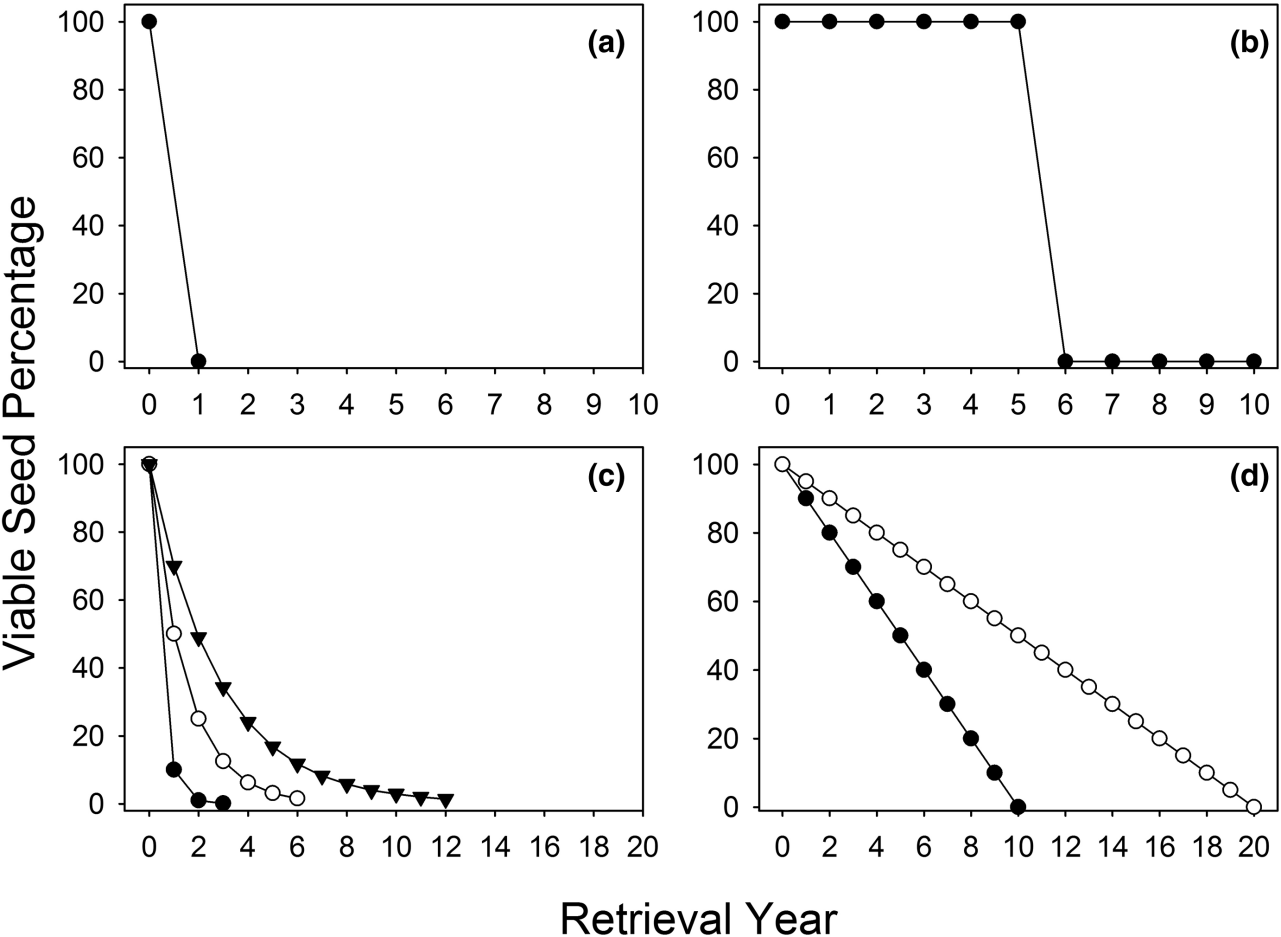
Seed bank depletion models for different patterns of seed persistence in soil seed banks: (a) Transient seed bank, all seeds germinate or lose viability within a year of entering the seed bank, (b) Cue‐responsive persistent seed bank, seeds lose dormancy and germinate in response to a specific cue after multiple‐year residence in the seed bank, (c) Short‐persistent seed bank, seeds lose dormancy and potentially germinate at a constant rate each year, resulting in a negative exponential seed bank depletion trajectory (black circles, 90% loss rate; white circles, 50% loss rate, black triangles, 30% loss rate), (d) Long‐persistent seed bank, a constant fraction of original seed cohort loses dormancy and potentially germinates each year, resulting in a linear seed bank depletion trajectory and age‐dependent annual depletion fractions (black circles, 10% of original cohort becomes nondormant each year, white circles, 5% of original cohort becomes nondormant each year).

Seed banks vary widely in their duration (Long et al., 2015). As seeds of a cohort either lose dormancy and germinate or suffer mortality from various causes, they are lost from the seed bank, and the shape of this loss trajectory can vary. Seeds that form transient seed banks either germinate or suffer mortality within the first year after production (Figure 1a). These species may have seeds that are nondormant and germinate in response to the first encounter with favorable conditions, or they may have cue‐responsive dormancy, for example, a dry after‐ripening or cold stratification requirement, that serves to time germination optimally within the year. Some species with persistent seed banks also have cue‐responsive dormancy (Figure 1b). Such species can persist in the seed bank for multiple years until the dormancy‐break cue, which is often associated with fire, triggers germination and seed bank depletion (Keeley, 1987). Other species have loss trajectories that are negatively exponential, with most seeds losing dormancy and germinating in the first few years (Figure 1c; Meyer & Kitchen, 1994). The model characteristic of this type of loss trajectory is a constant rate of dormancy loss of remaining seeds, which makes the loss rate independent of seed age. The loss rate can be dramatic or more gradual as shown in Figure 1c, but the absolute loss rate always slows with time. Finally, some loss trajectories are linear, with a relatively constant proportion of the original cohort of seeds losing dormancy and potentially germinating each year, making the seed loss fraction of the remaining seeds each year a function of seed age (Figure 1d). Such seeds are not cue‐responsive and lose dormancy at the same rate regardless of proximal environmental cues (Meyer et al., 2006), allowing for buffering of variable environmental conditions.

The shape of the seed bank loss trajectory for a species determines whether seeds will be present after extended periods of failed recruitment. However, for most plant species with seed banks, the residence time of individual seeds in the field seed bank and the trajectory of dormancy loss, germination, and loss of viability are unknown, largely because the studies that produce these kinds of data are both field‐intensive and long‐term. Various methods are used to study seed banks, with emergence from soil samples being a common method, but one that underestimates seed bank numbers (Abella et al., 2013; Gonzalez & Ghermandi, 2012) and does not provide information about seed age unless accompanied by carbon dating (Dalling & Brown, 2009), which is rarely used. Extraction (Gross, 1990) can provide more reliable numbers, but viability estimation can be difficult, as most efficient extraction methods negatively impact seeds, and again, there is no way to determine seed age. Species detection accuracy varies among methods as well (Price et al., 2010).

Seed retrieval studies are a labor‐intensive but highly informative method for studying seed banks. Placing bags of seeds in the soil in situ and retrieving replicates at regular intervals can provide good estimates of seed dormancy status, germination, and viability loss over time (Meyer et al., 2006), allowing researchers to quantify both seed bank duration and the shape of the seed bank loss trajectory.

Understanding seed bank dynamics can allow us to predict how a species might respond to climate change (Baskin & Baskin, 2022). Predicted changes in rainfall patterns, especially extended drought, will likely pose a risk to many seed‐banking species (Ooi, 2012). Inter‐annual variability in appropriately timed precipitation, and thus the length of time between successful recruitment years, will interact with seed bank duration to determine the likelihood of population extinction. Desert environments are highly variable and are among the biomes predicted to be most affected by climate change (IPCC, 2007). In deserts, mean precipitation can be so far below optimum that it would not permit population persistence. Stochasticity can be the primary factor maintaining a population, with years of above‐average precipitation replenishing the seed bank and therefore sustaining the population across series of years with “average” or lower precipitation that is inadequate for recruitment (Salguero‐Gomez et al., 2012; Van Buren et al., 2021). Predictions of increased stochasticity in the amount and timing of precipitation in desert environments (Maestre et al., 2012) raise the question of how seed banks of desert species will respond to climate change, and ultimately, whether or not these species will persist.

Here, we present a unique and very long‐term data set on seed bank dynamics in a rare desert gypsophile perennial, the Las Vegas bearpoppy (*A*. *californica*). Previously published research on the seed ecology of *A*. *californica* has established that seeds are largely dormant at dispersal (Pereira et al., 2021) and that a field seed bank is present (Abella et al., 2013; Megill et al., 2011; Winkel, 2004). We expand upon this work by presenting the results of our germination experiments, an in situ seed bank study, and a 15‐year field seed retrieval study.

Our research questions were: (1) What is the dormancy status of recently produced seeds? (2) Can conditions that seeds are expected to experience in the field allow recently dispersed seeds to break dormancy? (3) Does germination response differ among populations? (4) Is there a persistent field seed bank that remains even after a major germination event, and how is it spatially distributed? (5) What are the patterns of *A*. *californica* seed viability loss, dormancy loss, and field germination for multiple seed populations at two sites? We specifically wanted to establish the form of the seed bank loss trajectory for this species and to examine how seed population, site habitat, and inter‐annual weather variation influence seed bank loss rates.

## 2 MATERIALS AND METHODS

### 2.1 Study organism and habitat

**FIGURE 2:**
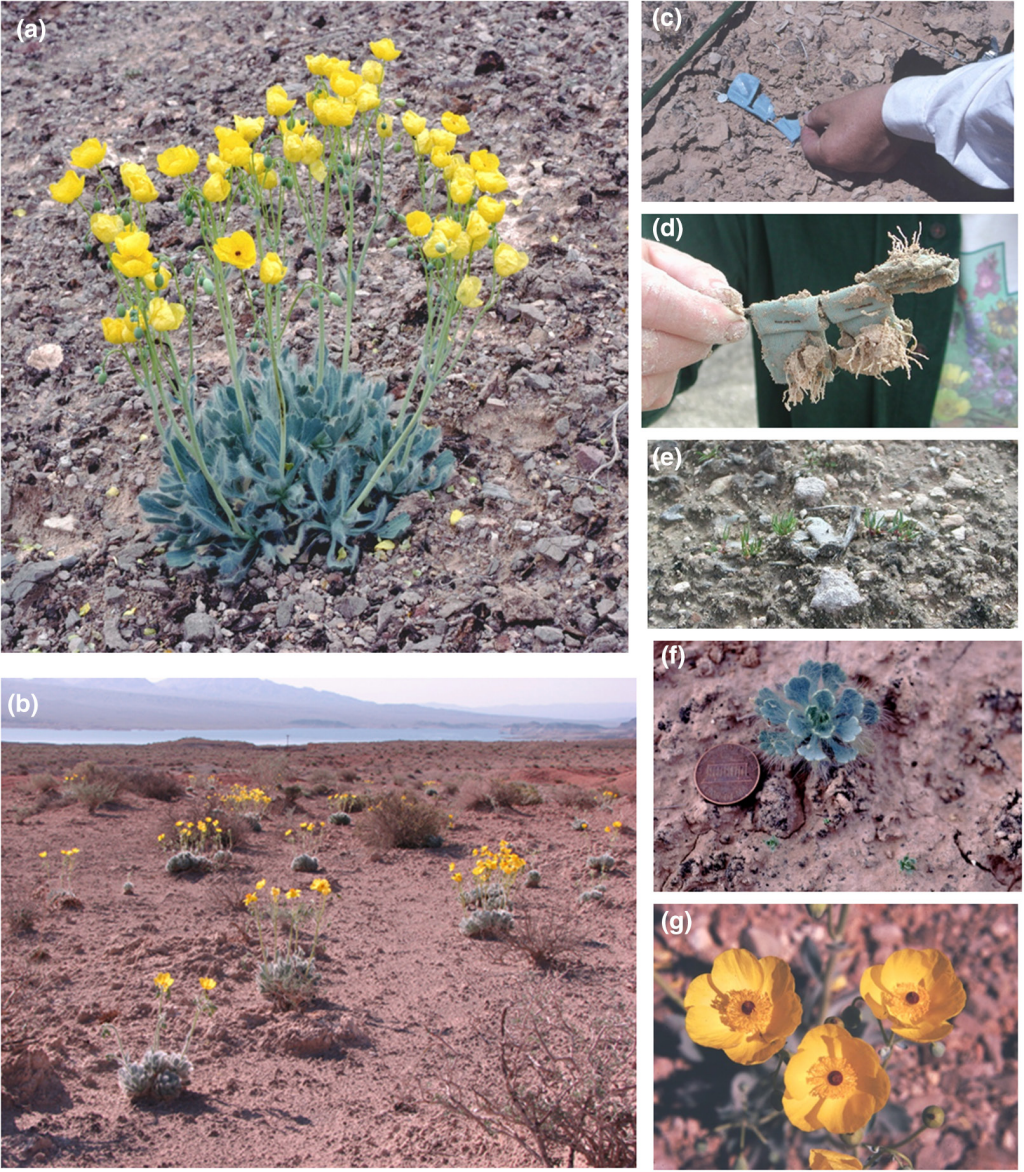
(a) *Arctomecon californica* (Las Vegas bearpoppy) adult in flower, (b) Gypsum habitat at the St. Thomas study site on Lake Mead National Recreation Area with flowering Las Vegas bear poppies, (c) Installing retrieval seed packets at the North Las Vegas Air Terminal in June 1995, (d) Retrieved seed packets in April 2005 showing radicle emergence from mass germination event, (e) New bearpoppy seedlings emerging from retrieval seed packets at North Las Vegas Air Terminal in April 2005, (f) Yearling bearpoppy plant surrounded by new seedlings at Rainbow Gardens in Apri1 1979, (g) Las Vegas bearpoppy flowers (All photos S. Meyer).

*Arctomecon californica*, the Las Vegas bearpoppy, is a rare herbaceous perennial plant of the Mojave Desert (Figure 2). The species is restricted to barren “badlands” soils high in gypsum or with other unusual chemical attributes (Meyer, 1980, 1986). It has lost much of its habitat, primarily due to urbanization (The Nature Conservancy, 2007). It currently occurs as fragmented, remnant populations in the Las Vegas Valley, larger populations around Lake Mead in Nevada and Arizona, and a few disjunct populations in the Lower Grand Canyon region. The species was recently petitioned for listing under the Endangered Species Act (Cornelisse & Tyler, 2019).

**FIGURE 3:**
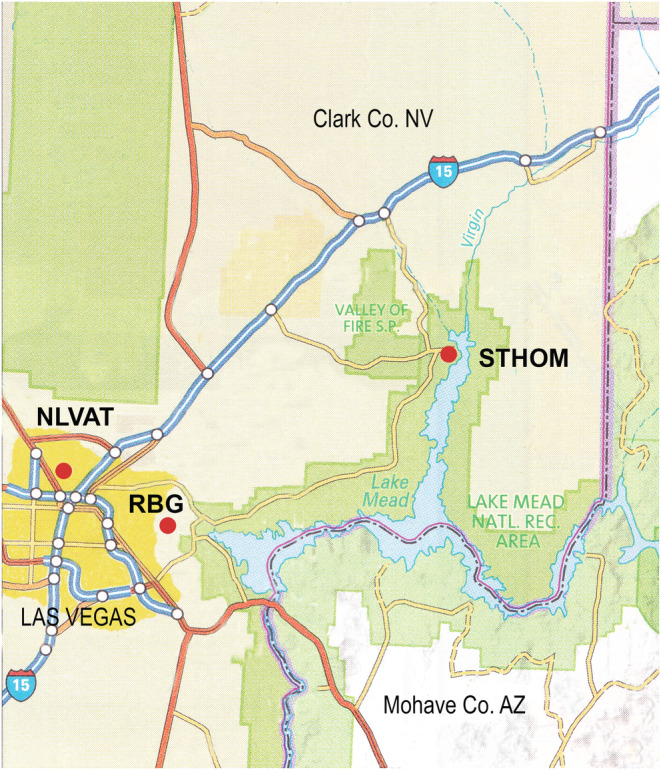
Map of the study sites (red dots) in southern Clark County, Nevada, USA: NLVAT (North Las Vegas Air Terminal, located within greater metropolitan Las Vegas, Nevada), RBG (Rainbow Gardens, located on USDI Bureau of Land Management land just east of Las Vegas, Nevada), STHOM (St. Thomas, located at the north end of Lake Mead National Recreation Area).

We chose two sites in different parts of the species range for the field experiments reported here (Table 1; Figure 3). The first site was a small Las Vegas bearpoppy preserve at the northeastern corner of the North Las Vegas Air Terminal in the Las Vegas Valley, while the second site was within a bearpoppy population on the road to the St. Thomas historic townsite near the north end of the Lake Mead National Recreation Area.

**TABLE 1:** Location and site information for the two sites included in field study. Climate data from PRISM explorer (https://prism.oregonstate.edu/explorer/).

Location and site information	Study site	
	North Las Vegas Air Terminal	St. Thomas road
Latitude/longitude	36.2085–115.1776	36.4482–114.3888
Elevation (m)	642 m	381 m
Geologic formation	Las Vegas formation	Muddy Creek Formation
Evaporite substrate origin	Pleistocene lakebed deposits/spring deposits	Tertiary lakebed deposits
Climate (1977–2021)		
Mean annual precipitation	110 mm	144 mm
CV^a^ annual precipitation	53%	47%
Mean Nov‐April precipitation	69 mm	90 mm
CV Nov‐April precipitation	74%	64%
Mean June–August precipitation	15.4 mm	24.1 mm
CV June–August precipitation	101%	74%
Mean annual temperature	20.1°C	20.6°C
Mean July max temperature	40.2°C	42.9°C

^a^ CV, Coefficient of variation = standard deviation/mean.

*Arctomecon californica* is a relatively short‐lived plant (known maximum life span of 6 years) that recruits from seed in sequences of unusually favorable years (Meyer, unpublished data). It has been thought to depend on a long‐lived seed bank for population persistence through sometimes long periods unfavorable for recruitment and survival (The Nature Conservancy, 2007).

**FIGURE 4:**
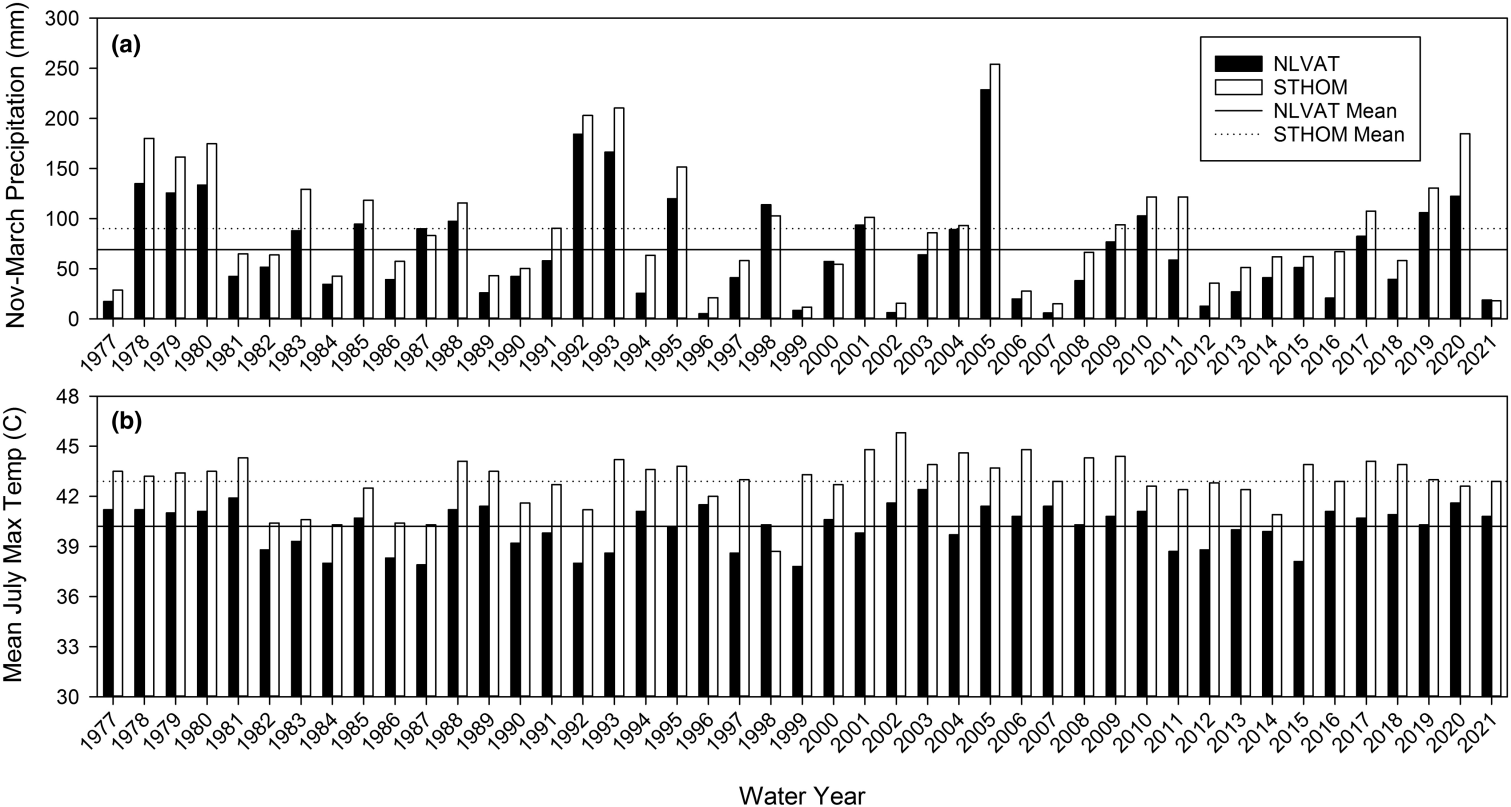
(a) Total winter–spring precipitation (November through March) and (b) mean maximum July temperature at the North Las Vegas Air Terminal and St. Thomas study sites from 1977 through 2021. Horizontal lines represent mean values at each site for the 1977–2021 period. Data from the Prism Explorer (https://prism.oregonstate.edu/explorer/).

Seeds of *A*. *californica* are known to exhibit morphophysiological dormancy (i.e., the embryo is immature at seed dispersal and also requires a physiological cue for dormancy break and embryo growth prior to germination). Wide variation among species exists in the conditions required to break morphophysiological dormancy (Baskin & Baskin, 2021). In some species, warm moist conditions break the physiological dormancy of the embryo, and embryo growth occurs under subsequent cold moist conditions. However, some seeds with morphophysiological dormancy can complete both dormancy break and embryo growth during moist chilling (Baskin et al., 1995). Spring germination of nondormant seeds in *A*. *californica* depends on winter/spring precipitation, which is highly variable among years (Figure 4a).

### 2.2 Seed germination study

**FIGURE 5:**
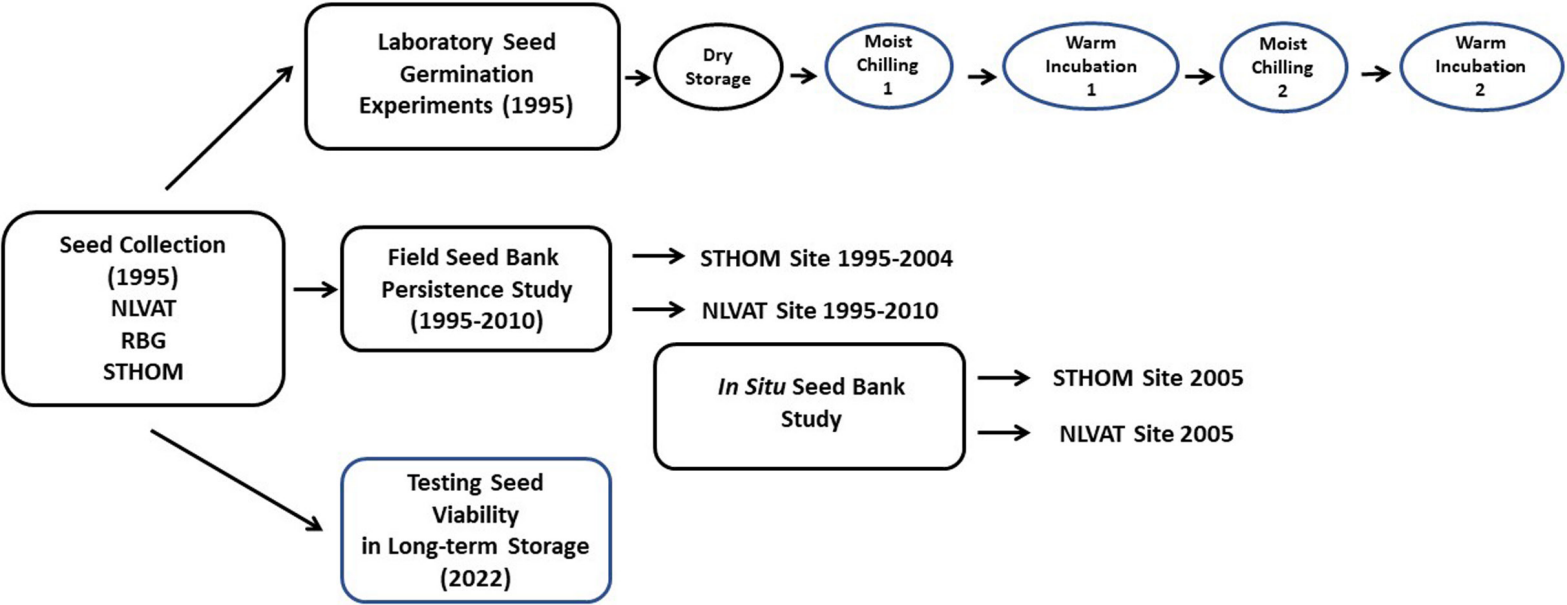
Design schematic showing the relationship of the five principal experimental components to each other and showing the treatment sequence included in the laboratory germination experiment.

The seed germination study was carried out in 1995 with bulk seed collections from ripe capsules of at least 30 individuals at field study sites at the North Las Vegas Air Terminal (NLVAT) and St. Thomas Road (STHOM), and also at Rainbow Gardens (RBG) on BLM land in June 1995 (Table 1, Figures 3 and 5). The germination experiment was carried out in the laboratory by placing recently harvested or experimentally stored seeds in Petri dishes on the surface of water‐saturated germination blotters, with subsequent incubation in temperature‐controlled chambers. The original experimental design included a total of 1032 experimental units (Petri dishes) and 462 treatment combinations. Each combination included either four replicate dishes of 25 seeds or two replicate dishes of 50 seeds as detailed below.

We determined in earlier experiments with a single 1985 seed population from Rainbow Gardens that a single warm moist stratification at 20°C plus a single short to intermediate cold moist stratification (4–12 weeks) at 2°C did not result in significant dormancy break for *A*. *californica* seeds previously stored under laboratory conditions for 3 years (germination ≤2%; Meyer, unpublished data). For this reason, we did not include a formal warm moist stratification pretreatment in the 1995 experimental design. Our treatments included the three seed populations (NLVAT, RBG, and STHOM), a series of 11 dry storage temperature‐by‐duration treatments, seven moist‐chilling duration‐by‐temperature combinations, and two postchilling incubation temperatures (Figure 5). One treatment sequence in our initial design included high‐temperature dry after‐ripening (50°C) followed by moist chilling on the logic that this combination was most likely to result in first‐year dormancy break in this spring‐emerging species.

The laboratory seed germination experiment was carried out in two steps: an original experiment (data not shown), and an additional experiment that added a re‐chill treatment as a continuation of the original experiment (Figure 5). The experimental design was factorial, with three seed populations (accessions), 11 storage duration‐temperature combinations, four moist‐chilling durations (0, 4, 8, and 12 weeks), two moist‐chilling temperatures (1°C, 5°C), and two postchilling incubation temperatures (5/15°C or 10/20°C for 12 h:12 h). Treatment combinations included either four Petri dish replicates (early treatments) or two replicates (remaining treatments).

For the storage treatments, seeds were stored at 20, 30, 40, and 50°C under unsealed dry conditions for 0–18 weeks. Shorter maximum durations were used at the two warmer temperatures based on our data from other species showing that prolonged storage at constant high temperatures could damage the seeds. Storage duration and storage temperature were therefore not combined in a full factorial design. After each chilling treatment, seeds were incubated at one of the two incubation temperatures for 2 weeks and scored for germination. In the earliest treatments, seeds were then immediately subjected to viability evaluation (AOSA, 2000); viability was consistently extremely high (≥99%).

After observing essentially no germination in any of the initial treatments, we decided to subject the later treatments to a second chilling experience after the 2‐week germination period. We chose a 12‐week cold stratification period for the re‐chilling treatment. This likely exceeds the maximum chilling duration that would be experienced in the field. After this second 12‐week cold stratification at the same temperatures as the original chilling treatments, the seeds were again incubated at the two original incubation temperatures for 2 weeks and scored for germination and viability. This second round of experimental treatments resulted in some dormancy break as reported below.

We have found that long‐term viability in dry storage is usually a necessary but not sufficient indicator of the ability of seeds to persist across years in field seed banks. We evaluated long‐term viability ex post facto for the seed lots used in germination and retrieval studies reported here by performing tetrazolium tests on four replicates of 50 seeds for each seed lot following 27 years of unsealed laboratory storage at room temperature (ca. 20°C). Seeds lose viability gradually over time in dry storage and this is reflected in a gradual diminution in respiration and the resulting red color after tetrazolium staining. As individual seeds lose viability at different rates, this results in a range of embryo color intensity. We took this into account by scoring seeds in three categories; viable (red or dark pink), marginal (pale pink), or nonviable (white). Seeds in the marginal category would probably not be able to produce seedlings, but the fact that they were still respiring indicates that they maintained viability longer than seeds in the nonviable (white) category.

### 2.3 In situ seed bank study

Plots were established in bearpoppy habitat at NLVAT and STHOM in March 2005 (Figures 3 and 5). Seed bank sample plots were set up within seedling establishment transects that were part of a separate study, therefore, seedling data are not presented here. Seedling establishment transects were located within areas where living or dead adult bearpoppies and gypsum soils were visible.

At NLVAT, 10 transects used for seed bank sampling were established across the study site. Quadrats were located at distances along the transect lines determined by random numbers generated using a stopwatch (Elzinga et al., 1998) and were designated only if seedlings were found within the randomly located 4 dm^2^ quadrat. At STHOM, the search protocol was different due to lower seedling densities. The entire area within 2 m on one side of each of the five transects was searched for seedlings. When seedlings were located, 4 dm^2^ quadrats were designated around them, and these plots were used for both the seedling study and the seed bank study. The transects were laid out in parallel at each site, but spacing and length of transects at each site were constrained by the requirement that there be suitable soils and evidence of bearpoppy occupation within each transect. This resulted in variable inter‐transect spacing and transect length. A paired seed bank sample was taken just outside of each seedling quadrat using a cylindrical soil can 6 cm in diameter and 4 cm in depth. Samples were lifted into labeled paper collection bags with the aid of a mason trowel. Sample size was 247 for NLVAT and 294 for STHOM. Samples were returned to the laboratory for sieving and seed extraction. It was relatively easy to visually identify the 2‐ to 3‐mm‐long jet‐black seeds of *A*. *californica* in the white soils where they occur. Seeds in each sample were counted and tested for viability using tetrazolium staining.

### 2.4 Seed retrieval study

Our seed retrieval study was conducted with the same bulk seed collections used in the germination experiments. It was installed at two sites (NLVAT and STHOM) and carried out over 15 years (1995–2010; Figure 5). Seeds were separated into sets of approximately 100 using a volume estimate and placed along with identification tags in 3 × 3 cm flat packets made of nylon mesh, which were folded over and stapled (Figure 2c). The seeds were returned to the field within 3 days of harvest. For each replicate, packets of each seed collection were buried as a set at approximately 1‐cm depth by lifting and then replacing the surface lichen crust with a mason trowel. Packet groups were installed in three linear blocks at each site with 20 packet sets at approximately 0.5 m spacing per block.

Starting in early summer 1996, seed packets at NLVAT were retrieved yearly through 2010, for a total of 15 retrieval dates. Retrievals at STHOM were discontinued in 2004, when ground squirrels destroyed the seed packets. On each retrieval date except for the final date, one set of seed packets from each block was excavated and taken to the laboratory for processing. On the final date, all remaining seed packets at NLVAT were retrieved. For each packet, any recently germinated seeds were counted and removed, and the remaining seeds were incubated at a near‐optimum temperature (5/15°C) for 4 weeks. Seeds that germinated during this period were scored as germinable (i.e., nondormant). Remaining ungerminated seeds at the end of the incubation period were then evaluated for viability using tetrazolium and were scored as nonviable, viable and dormant, or empty and presumed germinated in the field.

### 2.5 Statistical analyses

Laboratory germination data were analyzed as binomial data using PROC GLIMMIX in SAS (V.9.4, SAS Institute, Cary NC) in the trials/events format with germination fraction (number germinated/total number) as the response variable. This format provides analysis for a binomial response variable using the logit‐link function and the residual degrees of freedom method by default. Predictor variables for analysis as presented here included population, dry storage treatment, and incubation temperature. Initial chilling temperature and duration and re‐chilling temperature had inconsistent minor effects on germination outcomes in preliminary data exploration and were excluded from the present analysis.

In situ seed bank density (count) data were analyzed using SAS Proc GLM with population as the main effect and with transect nested within population. Viable seed fraction was analyzed using the same model as described above in SAS Proc GLIMMIX for binomial data. Viable seed proportion was calculated only for samples that had seeds present.

Seed retrieval data were first analyzed using SAS Proc REG for each seed population by site combination. For each combination, regression analysis was performed for the variables dormant seed fraction and viable (dormant plus nondormant) seed fraction. This analysis provided the slope of linear decline in seed dormancy and seed viability for each seed population at each site and also an estimate of the number of years to zero seed dormancy or viability, at the point on the time axis when dormancy or viability was projected to be zero.

Seed retrieval data were also analyzed using the GLIMMIX procedure in SAS. We used only the first 8 years of data from NLVAT in this analysis to make it directly comparable to the STHOM data set, which included only 8 years as previously explained. Block effects were not significant in this or any other analysis and were not included in the final models. Predictor variables included site (NLVAT and STHOM) and population of origin (STHOM, RBG, and NLVAT) as class variables and year as a continuous variable in the analysis of covariance. This enabled us to examine the effects of site, seed population, and their interaction on seed dormancy and viability loss rates. Field germination was estimated as the sum of recently germinated seeds (radicles evident) and empty seed coats. Field germination patterns were examined graphically but were not formally analyzed.

## 3 RESULTS

### 3.1 Seed germination study

In the original factorial experiment, the mean germination percentage across all seed populations and treatments and for each seed population by storage treatment combination was less than 1%, indicating a very high level of primary dormancy for recently produced seeds regardless of high‐temperature after‐ripening or moist chilling. Of 462 total treatment combinations, 428 had zero germination. The maximum germination percentage in any treatment combination was 5% (data not shown).

**FIGURE 6:**
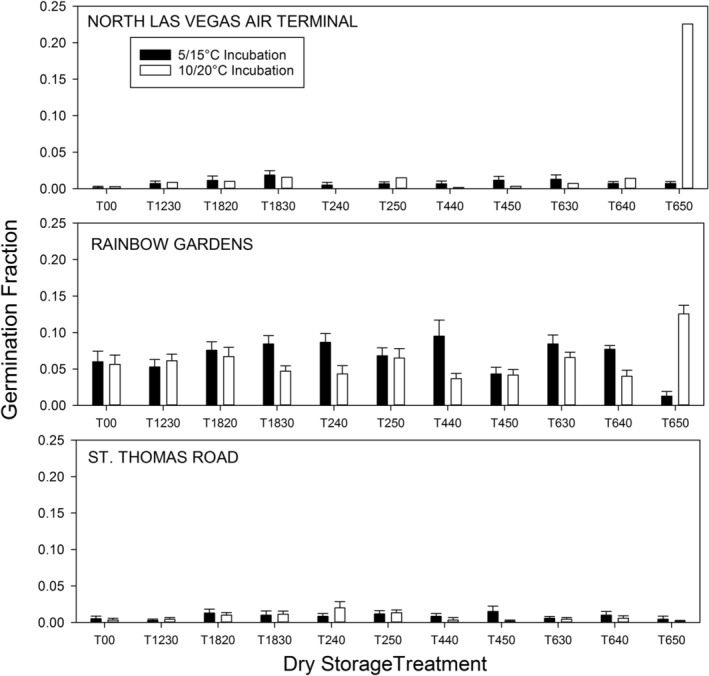
Seed germination fraction for three populations of *Arctomecon californica* seeds after dry storage at different temperatures and for different durations followed by chilling treatments, 2‐week incubation at one of two temperatures, re‐chilling for 12 weeks, and 2‐week post–re‐chilling incubation at the same two temperatures. Storage treatments are coded by duration in weeks followed by storage temperature (e.g., T1230 = 12 weeks at 30°C; T00 = seeds not stored prior to initiation of the experiment).

The 12‐week re‐chill treatment following storage, chilling, and incubation caused a major increase in germination for some storage treatment × seed population combinations, with both population (*F* = 169.05, *p* < .0001) and storage treatment (*F* = 3.31, *p* < .0003) highly significant (Table 2). There was little effect of the re‐chill on the St. Thomas Road seed population (STHOM), which still had a mean germination of ≤1% even after re‐chill (Figure 6). For the Rainbow Gardens seed population, mean germination increased from ≤1% to 6% following re‐chill. It showed little variation on average across storage treatments (4.3%–7.3%, Figure 6). The NLVAT seed population showed a strikingly different response. Its mean germination was uniformly low (<2%) across 10 of the 11 storage treatments, but with an initial long dry storage treatment at high temperature (T650 or 6 weeks at 50°C), mean germination increased to an average of 11.6% (Figure 6).

**TABLE 2:** Analysis of variance for germination proportion from the laboratory germination experiment using SAS 9.4 Proc GLIMMIX for binomial data and a completely randomized design. Main effects were seed population, dry storage treatment and postchilling incubation temperature. Chilling temperature and duration were determined to have minor and inconsistent effects on the response variable and were excluded from the final model.

Treatment effect	df	*F*‐Value	*p*‐Value
Seed population	2/870	169.05	<.0001
Storage treatment	10/870	3.31	.0003
Incubation temperature	1/870	0.00	.9714
Seed population × storage treatment	20/870	3.51	<.0001
Seed population × incubation temperature	2/870	0.60	.5467
Storage treatment × incubation temperature	10/870	3.06	.0008
Seed population × storage treatment × incubation temperature	20/870	1.77	.0198

The other striking effect of the re‐chill experiment was the significant three‐way interaction among population, storage treatment, and incubation temperature (Table 2; Figure 6). The STHOM seed population again showed germination <2% in response to any treatment. The RBG seed population showed low but consistent germination across storage treatments as mentioned earlier with no significant differential response to incubation temperature after 10 of 11 storage treatments. However, after 6 weeks at 50°C and 12‐weeks re‐chill, germination was 1% at 5/15°C but 13% at 10/20°C, a significant difference. The NLVAT population was similar to the St. Thomas population across 10 of the storage treatments, with germination <2%. This was also true after 5/15°C incubation following 6 weeks at 50°C, but at 10/20°C incubation temperature NLVAT seeds germinated to a mean of 23% across all initial chilling treatments, and to a high of 34% in the best treatment combination, by far the highest germination percentage achieved. These results are hard to interpret ecologically, but they show that the three seed populations had very different responses to experimental treatments. They also show that high‐temperature after‐ripening for 6 weeks at 50°C followed by chilling, warm incubation, and re‐chilling had a uniquely powerful effect on germination at the higher incubation temperature, and this effect differed by population (Figure 6).

**FIGURE 7:**
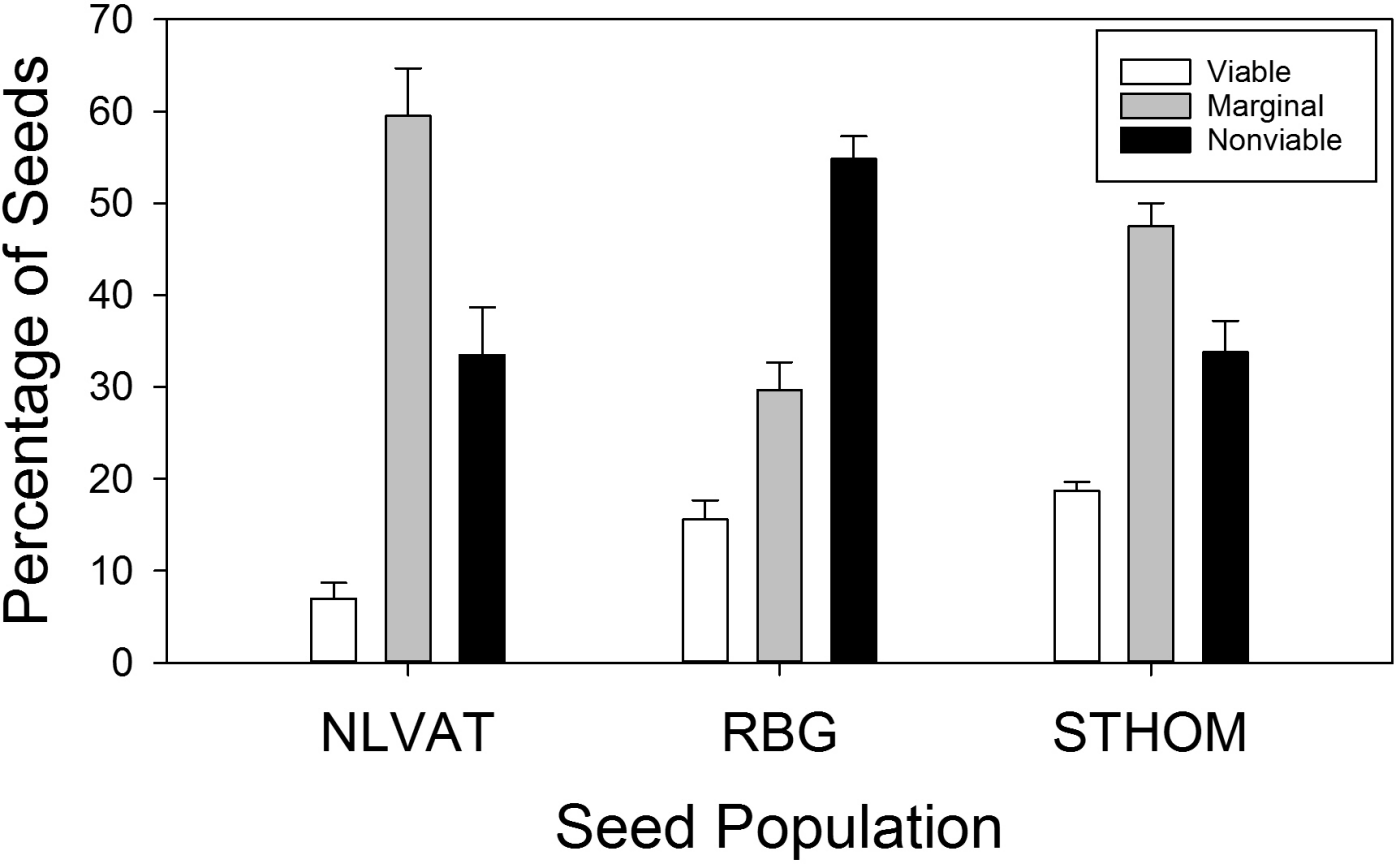
Seed viability after 27 years (1995–2022) of unsealed storage at laboratory temperature (ca. 20°C) for three seed populations used in the germination and seed retrieval experiments. Error bars are standard error of the mean based on four replicates of 50 seeds for each population.

Viability of seeds stored in the lab for 27 years was still surprisingly high even after prolonged laboratory storage (Figure 7). Tetrazolium testing showed that an average of 14% of the seeds were still clearly viable. In addition, almost half the seeds overall were scored as “marginal,” with only about 40% scoring as clearly not viable. Seeds in the “marginal” category were likely viable in the recent past, so it appears that the viability of laboratory‐stored *A*. *californica* seeds can remain quite high even after 20 years.

### 3.2 In situ seed bank study

**FIGURE 8:**
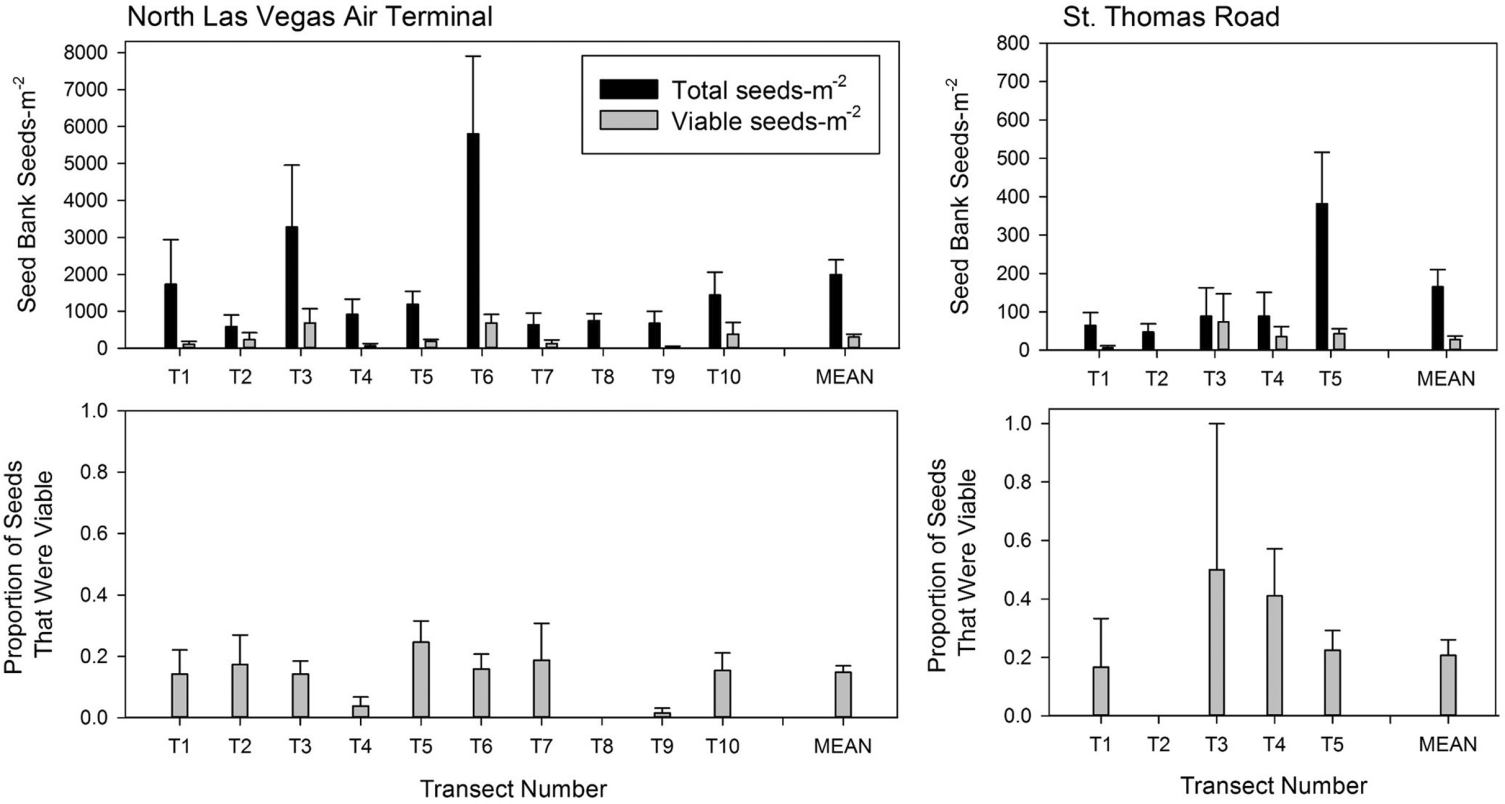
Total seed density, viable seed density, and fraction of seeds that were viable and presumed dormant in the soil seed bank at two sites (North Las Vegas Air Terminal and St. Thomas Road) the summer after a major germination event in 2005. Samples were collected along transects at the two sites where seedling data were also collected. Note scale difference in density at the two sites.

Our in situ seed bank study documented the density and viability of the seeds in the seed bank at NLVAT and STHOM after the mass germination event of spring 2005. First, we found an order of magnitude difference in seed density between the two sites, as indicated by the scale differences between the two graphs (Figure 8). Analysis of variance showed a highly significant difference in seed density between sites (Table 3). NLVAT was almost 10 times higher for both total seed density and viable seed density versus STHOM (310 vs. 28 viable seeds–m^−2^). There were also highly significant differences among transects within sites for total seed density, indicating a patchy distribution of seeds. Seed bank density also varied radically from sample to sample within transects, as evidenced by large error bars. Viable seed fraction was slightly higher for STHOM vs. NLVAT (bottom panel of Figure 8; 0.207 vs. 0.169), but this difference was not significant. Also, differences in viable seed fraction were not significant for transects within sites. In this study, seeds that were not viable were primarily represented by empty seed coats of previously germinated seeds, not seeds that had simply lost viability in the seed bank, suggesting that most loss from the seed bank was through germination. This study showed that viable and presumably dormant seeds could be present at relatively high density in field seed banks even after a mass germination event in response to exceptionally high winter–spring precipitation in 2005 (Figure 4a).

**TABLE 3:** Analysis of variance (SAS 9.4 Proc GLM) for the summer 2005 in situ seed bank study at North Las Vegas Air Terminal and St. Thomas. Viable seed proportion using SAS Proc GLIMMIX for binomial data was calculated only for samples that had seeds present.

Variable	Effect	df	*F*	*P*
Seeds per m^2^	Site	1526	15.15	.0001
	Transect (site)	13,526	3.19	.0001
Viable seeds per m^2^	Site	1526	7.74	.0056
	Transect (site)	13,526	1.58	.0862
Proportion of seeds viable	Site	1185	3.42	.0659
	Transect (site)	13,185	1.12	.3437

### 3.3 Seed retrieval study

**FIGURE 9:**
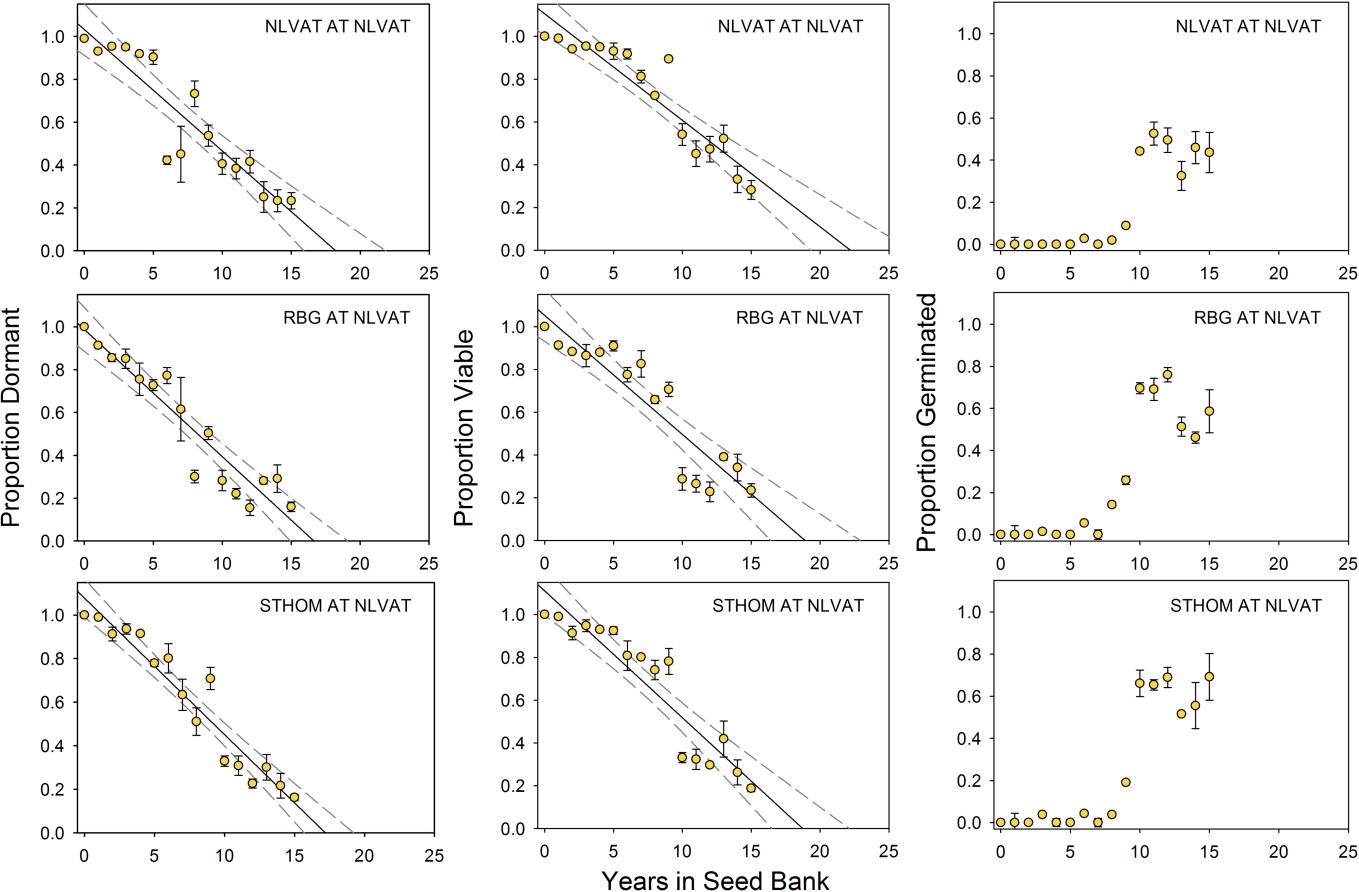
Results of a 15‐year (1995–2010) seed retrieval (artificial seed bank) study with seed populations from North Las Vegas Air Terminal, Rainbow Gardens, and St. Thomas Road conducted at the North Las Vegas Air Terminal study site. Error bars are standard error of the mean, solid lines are from linear regression analysis, and dashed lines represent 95% confidence intervals. See Table 4 for regression analysis.

Our seed retrieval experiment documents the rates of seed dormancy and viability loss and field germination events over 15 years. The set of graphs on the left‐hand sides of Figures 9 (NLVAT site) and 10 (STHOM site) illustrates the linear decrease in the proportion of dormant seeds over time at the two retrieval study sites. All R^2^ values for these regressions have significant levels of *p* < .0001 (Table 4).

**TABLE 4:** Regression equations for viable seed proportion (PVIABLE) and dormant seed proportion (PDORM), regressed on year for each of three populations and two sites. Probability of significance is *p* < .0001 for all regression equations. The slope of each equation is equivalent to the rate of dormancy loss or viability loss (i.e., the estimated fraction of total seeds that lose dormancy or viability in a year). NLVAT, North Las Vegas Air Terminal, RBG, Rainbow Gardens, STHOM, St. Thomas. See Figures 9 and 10 for a graphical representation of the dormant, viable, and germinated seed proportions during the course of the study.

Population	Site	Variable	df	Slope	Intercept	*R* ^2^_adj_	Projected years to zero
NLVAT	NLVAT	PVIABLE	54	−0.054	1.132	.8105	21.0
NLVAT	NLVAT	PDORM	54	−0.057	1.085	.7855	19.0
RBG	NLVAT	PVIABLE	55	−0.057	1.050	.7677	18.4
RBG	NLVAT	PDORM	55	−0.058	0.971	.8023	16.7
STHOM	NLVAT	PVIABLE	53	−0.063	1.124	.8408	17.8
STHOM	NLVAT	PDORM	53	−0.064	1.082	.8872	16.9
NLVAT	STHOM	PVIABLE	26	−0.053	1.095	.7265	20.7
NLVAT	STHOM	PDORM	26	−0.081	1.120	.7457	13.8
RBG	STHOM	PVIABLE	26	−0.041	0.988	.6355	24.1
RBG	STHOM	PDORM	26	−0.075	1.005	.7751	13.4
STHOM	STHOM	PVIABLE	25	−0.047	1.058	.6668	22.8
STHOM	STHOM	PDORM	25	−0.073	1.060	.6861	14.6

**FIGURE 10:**
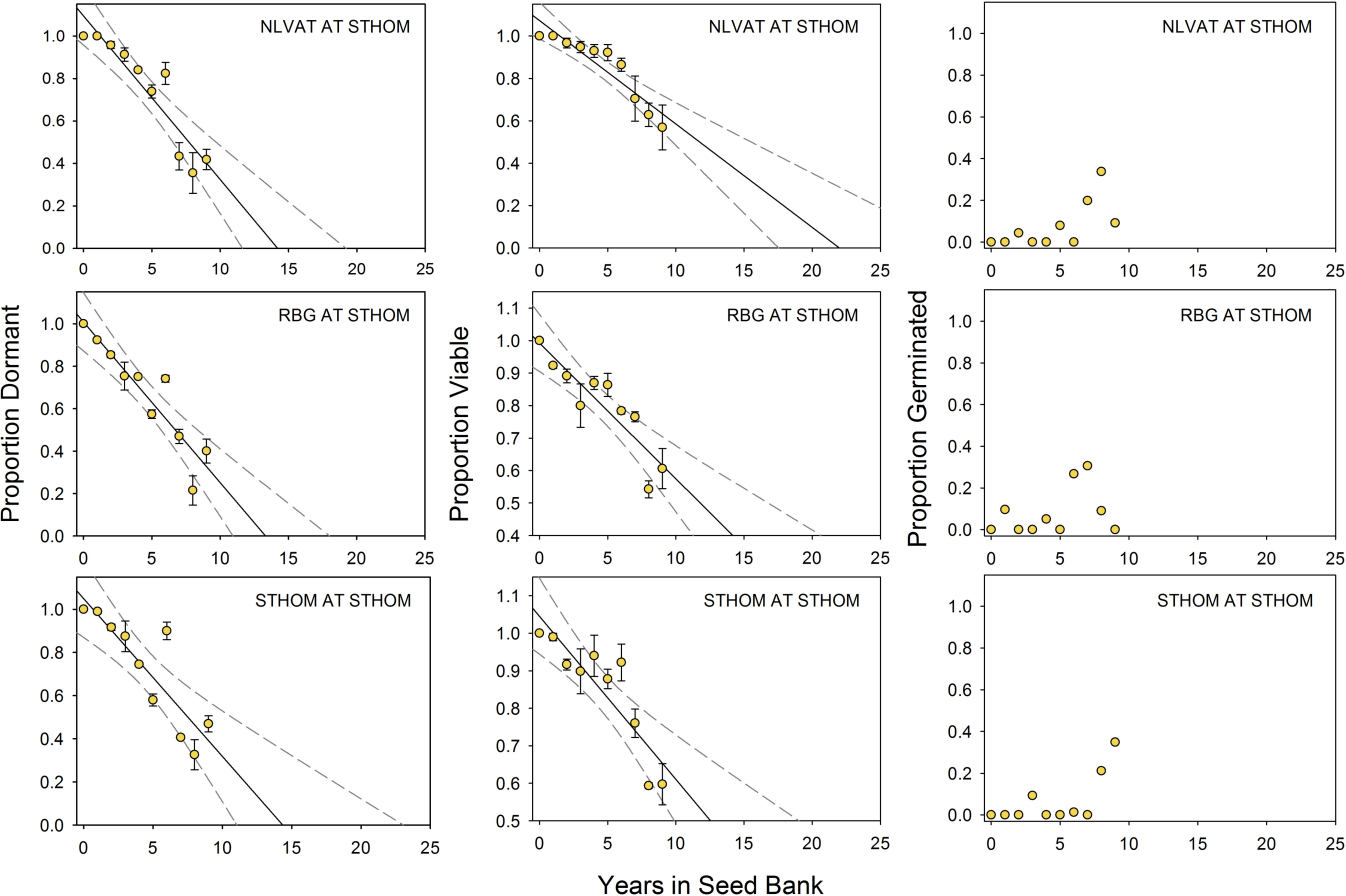
Results of a 9‐year (1995–2004) seed retrieval (artificial seed bank) study with seed populations from North Las Vegas Air Terminal, Rainbow Gardens, and St. Thomas Road conducted at the St. Thomas Road study site. Error bars are standard error of the mean, solid lines are from linear regression equations, and dashed lines represent 95% confidence intervals. See Table 4 for regression analysis.

The populations had slightly different rates of dormancy loss, with seeds from NLVAT losing dormancy at a slightly slower rate and those from RBG losing dormancy more quickly during the first few years (Population *F* = 20.16, *p* < .0001, Table 5). Site also affected rates of dormancy loss with seeds staying dormant significantly longer at NLVAT than at STHOM (Site *F* = 7.23, *p* < .0080, Table 5). For all population/site combinations, the proportion of seed that was dormant started at 1 (100%) in 1995 and decreased approximately linearly across the years of the study, with 10–20% of seeds still dormant after 15 years at NLVAT (Figures 9 and 10). At STHOM, retrievals were wrecked by ground squirrels after 8 years as mentioned earlier, but patterns were consistent across sites.

**TABLE 5:** Results of analysis of covariance (ANCOVA) for viable seed proportion and dormant seed proportion using SAS Proc GLIMMIX for binomial data. The analysis includes *Arctomecon californica* retrievals at North Las Vegas Air Terminal and St. Thomas for the period 1995–2004, the period during which retrievals were possible at both sites. Site and seed population were class variables while retrieval year was the continuous variable in ANCOVA.

Effect	df	Viable seed proportion		Dormant seed proportion	
		*F*‐Value	*p*‐Value	*F*‐Value	*p*‐Value
Site	1/151	40.8	<.0001	7.23	.0080
Seed population	2/151	24.1	<.0001	20.16	<.0001
Year	1/151	1127	<.0001	2354	<.0001
Year × site	1/151	40.9	<.0001	7.32	.0076
Year × seed population	2/151	24.0	<.0001	20.08	<.0001
Site × seed population	2/151	6.21	.0026	0.45	.6412
Year × site × population	2/151	6.22	.0025	0.45	.6392

The proportion of viable seeds over time is shown in the center columns of Figures 9 and 10. This fraction of total seeds includes dormant viable and nondormant viable seeds. Loss of viability over time was linear (*p* < .0001); however, the buildup of nondormant, viable seeds in the seed bank can be seen over the first 10 years of the study (which were relatively dry), followed by a drop in the number of viable seeds with the mass germination event in 2005, when all nondormant seeds in the packets germinated. It also shows the subsequent buildup of nondormant, viable seeds as seeds lost dormancy without germinating through the end of the study (Figures 9 and 10). As with dormancy loss, viability loss rates differed among populations (Population *F* = 24.1, *p* < .0001, Table 5) and sites (Site *F* = 40.8, *p* < .0001, Table 5).

Our last response variable was the approximate proportion of seeds that field‐germinated, as measured by the presence of recently germinated seeds and empty seed coats in the packets. These estimates are not as accurate as those involving viable seeds, but they can document germination events. They are shown across the right‐hand panels of Figures 9 and 10. The germination event of 2005 in response to the highest winter–spring precipitation in the 44‐year record (Figure 4a) is clearly visible as a jump in the field‐germinated fraction in Figure 9 for the NLVAT site. For STHOM, there appears to be some germination in years 6, 8, and 9 (Figure 10). These correspond to 2001, 2003, and 2004, years with average or above‐average winter–spring precipitation (Figure 4a).

Our regression equations allowed us to estimate the number of years it would take for all seeds to lose dormancy or viability (projected years to zero, Table 4). This tells us how long it would take for the seed bank to disappear if there were no seed production events to replenish it. For the different population/site combinations, time to zero dormant seed projections ranged from 13.4 years for RBG seeds at STHOM to 19.0 years for NLVAT seeds at NLVAT. Time to zero viable seed projections varied from 17.8 years for STHOM seeds at NLVAT to 24.1 years for RBG seeds at STHOM (Table 4). Seeds on average lost dormancy more quickly at STHOM than at NLVAT (average projected years to zero dormant seeds: 13.9 years for STHOM, 17.5 years for NLVAT) but lost viability more quickly at NLVAT (average projected years to zero viable seeds: 22.5 years for STHOM, 19.1 years for NLVAT). The longer period of viability at STHOM is partially an artifact of the shorter experimental duration there, as the retrieval experiment was destroyed before the 2005 germination event removed a sizeable fraction of viable seeds. However, these differences are significant even when the two sites are compared across the same number of retrieval years (Table 5; 1995–2004, site‐by‐year interactions for dormancy loss and viability loss).

For all population/site combinations, projected years to zero is lower when based on dormancy than when it is based on viability. This is because nondormant, viable seeds can persist in the seed bank during dry periods, extending seed bank survival. We propose that a reasonable general estimate for seed bank longevity based on viability loss over time for *A*. *californica* is approximately 20 years.

## 4 DISCUSSION

### 4.1 Germination study

The first stage of our germination experiment confirmed the hypothesis that *A*. *californica* seeds possess cue‐nonresponsive dormancy when recently harvested, as a series of treatments including only a single period of storage at summer temperatures followed by a single period of moist chilling and subsequent incubation at spring temperature produced minimal germination response. These treatments were designed as an approximate simulation of what seeds would experience during their first year following dispersal in early summer. However, we learned very little about what conditions would break primary dormancy or be conducive to the germination of nondormant seeds.

We decided to re‐chill the seeds after their first incubation period to try to elicit higher germination percentages. While this treatment does not clearly address any mechanism of dormancy break in the field, it did result in differentiation among the three seed populations. This means that a laboratory or field experiment with a single seed population is not generalizable across the species, which is consistent with findings from other species (Farooq et al., 2021; Meyer et al., 1995). Whether these differences are the result of selection on genetic variation or are maternal effects due to differences in seed ripening conditions is not known. Dormancy levels are known to be influenced by conditions during the period of seed maturation (Phillipi, 1993; Zhang et al., 2017).

One hypothesis for the effect of the re‐chill in increasing germination percentage is that the two‐week warm incubation period that preceded the re‐chill could have provided an opportunity for the physiological dormancy break necessary for subsequent embryo maturation and physiological response to chilling. It is possible that high‐temperature after‐ripening is a precondition for this effect during warm stratification and consequently was a precondition for the strong observed response to the re‐chill treatment following the 6 weeks at 50°C treatment, at least for the North Las Vegas Air Terminal seed population. To our knowledge, high‐temperature dry after‐ripening as a component of dormancy break for seeds with morphophysiological dormancy has not been previously reported. It will require more detailed experimental work to determine whether this is the correct explanation for the pattern we observed.

The germination responses following treatments preceded by high‐temperature after‐ripening are likely the most ecologically relevant for this species, which disperses its seeds in early summer into an environment where summer soil surface temperatures can exceed 60°C (Meyer, unpublished data). Studies have shown that some seeds can withstand surprisingly high temperatures (Ooi et al., 2009) and also maintain viability despite severe desiccation (Copete et al., 2021). *Arctomecon californica* seeds are clearly able to tolerate such hot, dry summer conditions. The fact that seeds lost dormancy more quickly at St. Thomas, which has hotter summers than North Las Vegas Air Terminal, provides circumstantial evidence that high‐temperature after‐ripening plays a role in dormancy loss (Table 4, Figure 4b; Figures 9 and 10).

Previous studies of Mojave Desert seed banks have also found that *A*. *californica* seeds are highly dormant (Abella et al., 2013; Pereira et al., 2021). Both our treatments and our results differed somewhat from those in the other laboratory study of germination requirements in *A*. *californica* (Pereira et al., 2021). However, warm (moderate) followed by cold temperature moist stratification appeared to be part of an effective dormancy‐breaking treatment in both studies. We obtained our highest germination percentage (>30%) after a combination of hot dry storage and cold–moderate–cold–moderate moist temperature stratification (Figure 5). The experiment of Pereira et al. (2021) lacked a high‐temperature storage treatment but also included alternating moderate and cold temperature moist stratification. The main difference was that the initial moist stratification treatment in their experiment was at a moderate temperature. In both experiments, it is assumed that morphophysiological dormancy has been broken in some proportion of the seeds, but it is unclear when the physiological dormancy break took place. In order to address the mechanism of dormancy break more precisely, an anatomical embryo study (Forbis & Diggle, 2001) would be required. A large majority of the seeds remained dormant in these experiments, despite the wide variety of treatments devised by the two research teams, suggesting that a large proportion of the seed dispersed each year has cue‐nonresponsive dormancy, allowing for long‐term seed bank persistence (Meyer, 2006).

### 4.2 In situ seed bank study

Our sampling to evaluate in situ seed banks was carried out in a year following a major germination event. Presumably, the number of viable seeds was lower than it would have been if samples had been taken following a series of years with low winter precipitation when there would have been a buildup of nondormant seeds. We found that seed density varied by a factor of 10 between sites and also varied widely both among transects and among samples within a transect within each site.

This patchy distribution of seeds in the seed bank corresponds to the results from Megill et al. (2011), who found the *A*. *californica* seed bank to be patchy at a site in North Las Vegas. They found that in sites with a high density of adults, seed distribution was correlated with adult plant distribution. Patchiness of seeds in the seed bank may also be related to patterns of seed predation (Cabin et al., 2000), and it is known that *A*. *californica* seeds are moved by ants (Megill, 2007). The patchiness of seeds in the soil highlights the importance of protecting large areas where *A*. *californica* populations are known to have existed in the past, as high seed densities can be concentrated in areas not evident by inspection.

### 4.3 Seed retrieval study

In the retrieval study, one objective was to establish the form of the seed bank loss trajectory for this species and to examine how seed population, site habitat, and inter‐annual weather variation influence rates of seed bank loss. Our data indicate that seeds can remain dormant and viable in the seed bank over many years regardless of field conditions, which varied widely throughout the course of our study (Figure 4). It clearly demonstrated that *A*. *californica* seed bank loss trajectories are of the linear form shown in Figure 1d. Once seeds become nondormant, they can also remain viable over multiple years until a germination‐triggering rainfall event occurs. Our laboratory germination study did not simulate what happens in the field, namely the effect of multiple years of after‐ripening, but it is clear from the field data that each year, another fraction undergoes dry after‐ripening and becomes either nondormant or chilling‐responsive. This annual transition of a small and relatively constant proportion of seeds into the cue‐responsive fraction through high‐temperature after‐ripening is similar to the pattern of dormancy loss in shadscale (*Atriplex confertifolia*; Meyer et al., 1998; Garvin & Meyer, 2003), another desert species with a persistent seed bank.

The *A*. *californica* seed retrieval study provided strong evidence that this species has a long‐lived seed bank due to cue‐nonresponsive seed dormancy in which only a small fraction of seeds (roughly 5%) become nondormant each year, allowing seed banks of this species to last up to 20 years without a seed production event. The retrievals allowed us to document that seeds of known age lost dormancy year by year, creating a buildup of nondormant seeds in the seed bank during the series of dry years between 1996 and 2005 (Figures 9 and 10). At St. Thomas, there was some germination between 2000 and 2005, but, especially at North Las Vegas Air Terminal, most of the germination occurred in 2005 (year 10). The jump in germination at year 10 is mirrored by the sharp decline in the proportion of viable seeds in the seed bank at North Las Vegas Air Terminal (Figure 9).

The difference in dormancy loss and germination patterns at the two study sites is likely related to climate differences (Table 1; Figure 4). St. Thomas has higher winter–spring precipitation on average, making a marginal germination‐triggering winter storm more likely to be effective there than at North Las Vegas Air Terminal (Figure 4a). In addition, summer air temperatures at St. Thomas are measurably higher than at North Las Vegas Air Terminal (Figure 4b), and this is probably even more true of seedbed temperatures. If dry after‐ripening is accelerated at high temperature, as is provisionally indicated in our germination experiment, we would expect seeds to become nondormant more quickly at St. Thomas. This difference in the dormancy loss trajectory at the two sites is readily observable in the retrieval data (Table 4; Figures 9 and 10).

Seeds sometimes have visible heteromorphism that allows for variation in germination behavior leading to bet‐hedging (Long et al., 2015). They can also have cryptic heteromorphism that allows for variation in the duration of dormancy (Venable, 1985). *Arctomecon californica* appears to have cryptic heteromorphism that allows individual seeds to differ in the duration of their cue‐nonresponsive dormancy. There is evidence for cryptic heteromorphism in its congener *A*. *humilis*, as seeds produced late in the season have lower dormancy levels than seeds produced during peak production (S. Meyer, unpublished data). We do not know the mechanism that is producing variation in dormancy duration within populations of *A*. *californica*, but a future study looking at dormancy levels among seeds produced at different times during the growing season might provide information.

## 5 CONCLUSIONS

Our seed bank persistence study provides strong evidence that sites known to have had plants of *A*. *californica* within the last 20 years should be considered viable populations, even if no plants have been present for many years. Given the pace of development in the Las Vegas Valley, it is easy to imagine that sites lacking any signs of a bearpoppy population might be considered unoccupied and not in need of protection. However, our results make it clear that locations that have had bearpoppy populations in the past likely contain viable seed banks, and therefore, viable (if invisible) populations. These sites need to be protected.

The precipitation record (Table 1; Figure 4a) shows clearly the high inter‐annual variation in winter–spring precipitation in this ecosystem and the very low mean values at these two sites. We also found a lack of first‐order temporal autocorrelation in winter–spring precipitation totals across years at both sites, indicating there is no way to predict precipitation in future years on a short time scale. Given that climate change is expected to increase both drought and variability in precipitation patterns in arid systems, understanding how seed dormancy influences extinction risk is crucial. Our data illustrate the cycle that *A*. *californica* seed banks have experienced throughout their history, with seeds produced in favorable years entering the seed bank, then losing dormancy at a slow, constant rate and creating a pool of nondormant seeds. These seeds can then germinate *en masse* when there is a year of exceptionally favorable winter precipitation. A key take‐home message from this work is that a population of *A*. *californica* can persist for at least 20 years without any aboveground plants. Whether this impressive life history strategy can maintain this species in the face of climate change depends on the length of future droughts or, conversely, on the frequency of well‐timed precipitation that allows for germination events followed by the survival of seedlings to adulthood and seed production.

Knowledge of seed bank dynamics alone is clearly not sufficient to address the impacts of climate change on plant demography. The seed ecology data sets presented here will be combined with additional data sets addressing recruitment, survival to reproduction, and adult lifespan and reproductive output in our upcoming population viability analysis for this species. We plan to use future climate predictions in conjunction with these demographic data sets to evaluate whether *A*. *californica* is likely to persist as temperature and precipitation patterns change.

## AUTHOR CONTRIBUTIONS

**Tara de Queiroz:** Conceptualization (supporting); data curation (supporting); funding acquisition (supporting); investigation (supporting); visualization (supporting); writing – original draft (lead); writing – review and editing (equal). **Susan E Meyer:** Conceptualization (lead); data curation (lead); formal analysis (lead); funding acquisition (lead); investigation (lead); visualization (lead); writing – original draft (supporting); writing – review and editing (equal).

## CONFLICT OF INTEREST STATEMENT

The authors declare no conflict of interest.

## Data Availability

Field and laboratory data sets that form the basis of the analyses in this manuscript are deposited in DRYAD at https://doi.org/10.5061/dryad.44j0zpcjj.
